# Supplementary material for: Koopman Invariant Subspaces and Finite Linear Representations of Nonlinear Dynamical Systems for Control
Source: PLoS One. 2016 Feb 26;11(2):e0150171. doi: 10.1371/journal.pone.0150171 (PMC4769143; doi:10.1371/journal.pone.0150171)
Supplement: S1 Appendix — (PDF) [file pone.0150171.s001.pdf]

Supporting Information for: Koopman Invariant Subspaces and Finite Linear Representations of Nonlinear Dynamical Systems for Control

Steven L. Brunton[1,*], Bingni W. Brunton[2], Joshua L. Proctor [3], J. Nathan Kutz[4],

1 Department of Mechanical Engineering, University of Washington, Seattle, WA 98195, United States
2 Department of Biology, University of Washington, Seattle, WA 98195, United States
3 Institute for Disease Modeling, Bellevue, WA 98004, United States
4 Department of Applied Mathematics, University of Washington, Seattle, WA 98195, United States

*** sbrunton@uw.edu**

Systems without Koopman-invariant subspaces that explicitly span the state

For any system with multiple fixed points, periodic orbits, or atrracting/repelling structures, there is no finite-dimensional Koopman invariant subspace that explicitly includes the state. This follows from the fact that these systems cannot be topologically conjugate to a finite-dimensional linear system with a single fixed point. It may, however, be possible to obtain a linearization that is valid in an entire basin of attraction of a single fixed point or periodic orbit of a complex system [1, 2]. Moreover, it may be possible to determine an invariant subspace spanned by Koopman eigenfunctions such that it is possible to invert these eigenfunctions to recover the states. Incorporating eigenfunction-based Koopman optimal control is an important avenue of future research, as it will open up Koopman optimal nonlinear control to a wider class of important problems.

Example: Logistic map

Consider the logistic map, given by:

$$x_{k+1} = r x_k (1 - x_k). \tag{1}$$

Naturally, the observable subspace must include x and x^2:

$$\mathbf{y}_k = \begin{bmatrix} x \\ x \end{bmatrix}_k \triangleq \begin{bmatrix} x_k \\ x_k^2 \end{bmatrix}. \tag{2}$$

Writing out the Koopman operator, the first row equation is simple:

$$\mathbf{y}_{k+1} = \begin{bmatrix} x \\ x^2 \end{bmatrix}_{k+1} = \begin{bmatrix} 2r & -r \\ ? & ? \end{bmatrix} \begin{bmatrix} x \\ x^2 \end{bmatrix}_k, \tag{3}$$

but the second row is not obvious. To find this expression, expand $(x_{k+1})^2$:

$$x_{k+1}^2 = \left(r x_k (1 - x_k)\right)^2 = r^2 \left(x_k^2 - 2x_k^3 + x_k^4\right). \tag{4}$$

Thus, we also need cubic and quartic polynomial terms to advance x^2. Similarly, these terms need polynomials up to sixth and eighth order, respectively, and so on, ad infinitum:

$$\begin{bmatrix} x \\ x^2 \\ x^3 \\ x^4 \\ x^5 \\ \vdots \end{bmatrix}_{k+1} = \begin{array}{c} \begin{matrix} x & x^2 & x^3 & x^4 & x^5 & x^6 & x^7 & x^8 & x^9 & x^{10} & \end{matrix} \\ \begin{bmatrix} r & -r & 0 & 0 & 0 & 0 & 0 & 0 & 0 & 0 & \cdots \\ 0 & r^2 & -2r^2 & r^2 & 0 & 0 & 0 & 0 & 0 & 0 & \cdots \\ 0 & 0 & r^3 & -3r^3 & 3r^3 & r^3 & 0 & 0 & 0 & 0 & \cdots \\ 0 & 0 & 0 & r^4 & -4r^4 & 6r^4 & -4r^4 & r^4 & 0 & 0 & \cdots \\ 0 & 0 & 0 & 0 & r^5 & -5r^5 & 10r^5 & -10r^5 & 5r^5 & -r^5 & \cdots \\ \vdots & \vdots & \vdots & \vdots & \vdots & \vdots & \vdots & \vdots & \vdots & \vdots & \ddots \end{bmatrix} \end{array} \begin{bmatrix} x \\ x^2 \\ x^3 \\ x^4 \\ x^5 \\ \vdots \end{bmatrix}_k .$$

It is interesting to note that the rows of this equation are related to the rows of Pascal's triangle, with the n-th row scaled by r^n, and with the omission of the first row:

$$\left[x^0\right]_{k+1} = \left[r^0\right] \left[x^0\right]_k. \tag{5}$$

The representation of the Koopman operator in a polynomial basis is somewhat troubling. Not only is there no closure, but the determinant of any finite-rank truncation is very large for $r > 1$. This illustrates a pitfall associated with naive representation of the infinite dimensional Koopman operator for a simple chaotic system. Truncating the system, or performing a least squares fit on an augmented observable vector (i.e., DMD on a nonlinear measurement) yields poor results, with the truncated system only agreeing with the true dynamics for a small handful of iterations.

Example: Nonlinear fixed point with a center manifold

Consider the simple nonlinear system with a single isolated fixed point at the origin:

$$\frac{d}{dt} x = x^2. \tag{6}$$

The Carleman linearization approach above would suggest that we augment the observable subspace with the quadratic polynomial $y_2 = x^2$, so that:

$$\begin{bmatrix} y_1 \\ y_2 \end{bmatrix} = \begin{bmatrix} x \\ x^2 \end{bmatrix}. \tag{7}$$

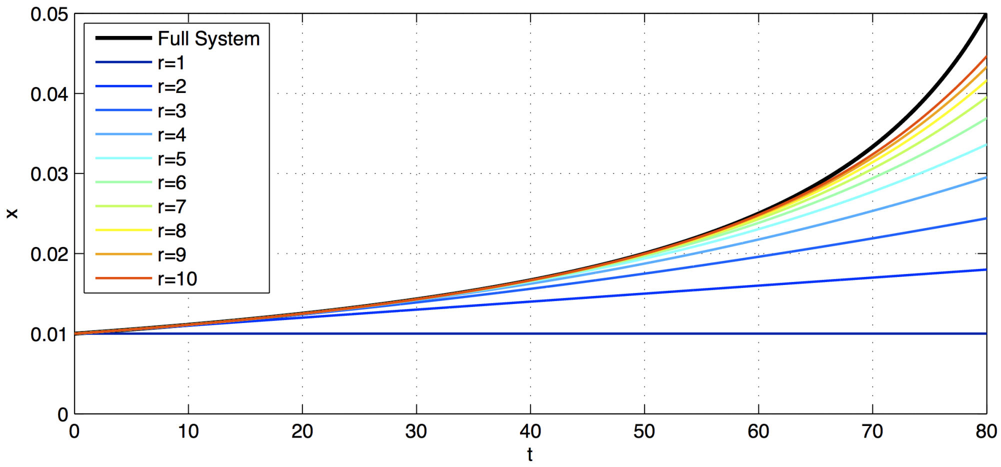

Figure S1. Illustration of Koopman linear system from Eq. (9) converging towards true solution as the rank of the truncation r is increased.

However, the expression for the time-derivative of y_2 requires higher polynomials in x:

$$\frac{d}{dt}y_2 = 2x\dot{x} = 2x^3. \tag{8}$$

Similarly, if we introduce $y_3 = x^3$, then $\frac{d}{dt}y_3 = 3x^2\dot{x} = 3x^4$, and so on. This results in an infinite Koopman expansion:

$$\frac{d}{dt}\begin{bmatrix} y_1 \\ y_2 \\ y_3 \\ y_4 \\ \vdots \end{bmatrix} = \begin{bmatrix} 0 & 1 & 0 & 0 & \cdots \\ 0 & 0 & 2 & 0 & \cdots \\ 0 & 0 & 0 & 3 & \cdots \\ 0 & 0 & 0 & 0 & \cdots \\ \vdots & \vdots & \vdots & \vdots & \ddots \end{bmatrix}\begin{bmatrix} y_1 \\ y_2 \\ y_3 \\ y_4 \\ \vdots \end{bmatrix} \quad \text{where} \quad \begin{bmatrix} y_1 \\ y_2 \\ y_3 \\ y_4 \\ \vdots \end{bmatrix} = \begin{bmatrix} x \\ x^2 \\ x^3 \\ x^4 \\ \vdots \end{bmatrix}. \tag{9}$$

Note that the determinant of any finite-rank truncation of the Koopman operator is 0, even though the system has finite-time blow up, as seen in Fig. S1. For this problem, it is possible to use eigenfunction coordinates to obtain a linear model in terms of an eigenfunction that may be inverted to recover the state (this is from a personal communication with C. W. Rowley):

$$\varphi(x) = e^{-1/x} \quad \implies \quad \frac{d}{dt}\varphi(x) = x^{-2}e^{-1/x}\dot{x} = \varphi(x). \tag{10}$$

Identifying eigenfunctions from data and using these linear models for control is a high-priority future direction.

Code 1. Koopman linear system corresponding to Fig. 3.

```
clear all, close all, clc
%% System
mu = -.05;
lambda = -1;
A = [mu 0 0; 0 lambda -lambda; 0 0 2*mu];  % Koopman linear dynamics
[T,D] = eig(A);
slope_stab_man = T(3,3)/T(2,3);  % slope of stable subspace (green)

%% Integrate Koopman trajectories
y0A = [1.5; -1; 2.25];
y0B = [1; -1; 1];
y0C = [2; -1; 4];
tspan = 0:.01:1000;
[t,yA] = ode45(@(t,y)A*y,tspan,y0A);
[t,yB] = ode45(@(t,y)A*y,tspan,y0B);
[t,yC] = ode45(@(t,y)A*y,tspan,y0C);

%% Plot invariant surfaces
% Attracting manifold $y_2=y_1^2$ (red manifold)
[X,Z] = meshgrid(-2:.01:2,-1:.01:4);
Y = X.^2;
surf(X,Y,Z,'EdgeColor','None','FaceColor','r','FaceAlpha',.1)
hold on, grid on, view(-15,8), lighting gouraud

% Invariant set $y_3=y_1^2$ (blue manifold)
[X1,Y1] = meshgrid(-2:.01:2,-1:.01:4);
Z1 = X1.^2;
surf(X1,Y1,Z1,'EdgeColor','None','FaceColor','b','FaceAlpha',.1)

% Stable invariant subspace of Koopman linear system (green plane)
[X2,Y2]=meshgrid(-2:0.01:2,0:.01:4);
Z2 = slope_stab_man*Y2;  % for mu=-.2
surf(X2,Y2,Z2,'EdgeColor','None','FaceColor',[.3 .7 .3],'FaceAlpha',.7)

x = -2:.01:2;
% intersection of green and blue surfaces (below)
plot3(x,(1/slope_stab_man)*x.^2,x.^2,'-g','LineWidth',2)
% intersection of red and blue surfaces (below)
plot3(x,x.^2,x.^2,'--r','LineWidth',2)
plot3(x,x.^2,-1+0*x,'r--','LineWidth',2);

%% Plot Koopman Trajectories (from lines 15-17)
plot3(yA(:,1),yA(:,2),-1+0*yA,'k-','LineWidth',1);
plot3(yB(:,1),yB(:,2),-1+0*yB,'k-','LineWidth',1);
plot3(yC(:,1),yC(:,2),-1+0*yC,'k-','LineWidth',1);
plot3(yA(:,1),yA(:,2),yA(:,3),'k','LineWidth',1.5)
plot3(yB(:,1),yB(:,2),yB(:,3),'k','LineWidth',1.5)
plot3(yC(:,1),yC(:,2),yC(:,3),'k','LineWidth',1.5)
plot3([0 0],[0 0],[0 -1],'ko','LineWidth',4)
set(gca,'ztick',[0 1 2 3 4 5])
axis([-4 4 -1 4 -1 4])
xlabel('y_1'), ylabel('y_2'), zlabel('y_3');
```

Code 2. Koopman operator optimal control (KOOC) example corresponding to Fig. 4.

```
clear all, close all, clc

mu = -.1;
lambda = 1;
tspan = 0:.01:50;
x0 = [-5; 5];

% LQR on linearized system
A = [-.1 0; 0 1];
B = [0; 1];
Q = eye(2);
R = 1;
C = lqr(A,B,Q,R);
vf = @(t,x) A*x + [0; -lambda*x(1)^2] - B*C*x;
[t,xLQR] = ode45(vf,tspan,x0);

% Koopman operator optimal control (KOOC); i.e., LQR on Koopman
    operator
A2 = [mu 0 0; 0 lambda -lambda; 0 0 2*mu];
B2 = [0; 1; 0];
Q2 = [1 0 0; 0 1 0; 0 0 0];
R = 1;
C2 = lqr(A2,B2,Q2,R);
% note that controller is nonlinear in the state 'x'
vf2 = @(t,x) A*x + [0; -lambda*x(1)^2] - B*C2(1:2)*x + [0; -C2
    (3)*x(1)^2];
[t,xKOOC] = ode45(vf2,tspan,x0);

%% Plot
figure(1)
subplot(1,3,1)
plot(xLQR(:,1),xLQR(:,2),'k','LineWidth',1.2);
hold on, grid on
plot(xKOOC(1:50:end,1),xKOOC(1:50:end,2),'r--','LineWidth',1.2);
xlabel('x_1'), ylabel('x_2')

subplot(1,3,2)
plot(tspan,xLQR,'k','LineWidth',1.2);
hold on, grid on
plot(tspan,xKOOC,'r--','LineWidth',1.2);
xlabel('t'), ylabel('x_k')
xlim([0 50])

JLQR = cumsum(xLQR(:,1).^2 + xLQR(:,2).^2 + (C*xLQR')'.^2)';
JKOOC = cumsum(xKOOC(:,1).^2 + xKOOC(:,2).^2 + (C*xKOOC')'.^2)';
subplot(1,3,3)
plot(tspan,JLQR,'k','LineWidth',1.2);
hold on, grid on
plot(tspan,JKOOC,'r--','LineWidth',1.2);
xlabel('t'), ylabel('J')
axis([0 50 0 500000])
legend('LQR','Koopman optimal control')
```

References

1. Williams MO, Kevrekidis IG, Rowley CW. A data-driven approximation of the Koopman operator: extending dynamic mode decomposition. Journal of Nonlinear Science. 2015;25(6):1307–1346.
2. Lan Y, Mezić I. Linearization in the large of nonlinear systems and Koopman operator spectrum. Physica D: Nonlinear Phenomena. 2013;242(1):42–53.
